# Data Modernization Opportunity: Alignment between US Public Health Data Policies and Public Health Ethics

**DOI:** 10.23889/ijpds.v9i5.2901

**Published:** 2024-09-18

**Authors:** Regen Weber-Fares, Fallon Cochlin, Hye-Chung Kum, Cason Schmit

**Affiliations:** 1 Texas A&M University; 2 Population Informatics Lab; 3 Program in Health, Law, and Policy

## Objective and Approach

Modernizing data sharing policies is key to fix deficiencies exposed by COVID-19. Decentralized US public health data collection, with varying state laws, challenges data sharing across governmental entities. Many data protection laws rest on bioethical principles that de-emphasize collective interests, creating challenges for public health data governance.

Here, we analyze US state public health data laws with WHO ethical guidelines, using a modified policy surveillance method to examine laws regulating data sharing between public health partners. We assess state law alignment with WHO guidelines for (1) public health data use, (2) research use, (3) emergency use, and (4) law enforcement use.

## Results

We found 34 states with laws explicitly allowing data sharing with federal public health agencies, 16 states permitting data sharing for research, 10 with provisions for emergencies, and 14 states permitting some data sharing for law enforcement.

## Conclusions

Efficient US public health data modernization needs policies that enable effective data sharing with proper justification and governance. The WHO offers guidance on ethically sharing sensitive data, but many US states' laws don't align with these guidelines, based on public health ethics. Instead, state laws often reflect bioethical standards, which are increasingly seen as unsuitable for managing population data in public health.

## Implications

Supportive policies are crucial for allowing population data to be used for social benefits. Policies that go against ethical guidelines and impose unnecessary obstacles to data sharing can hinder vital activities, like record linkage, that support public health efforts and collective benefits.

